# Liquid Metal Biomimicry: Bridging Fluidity and Biological Adaptability

**DOI:** 10.3390/biomimetics11070499

**Published:** 2026-07-16

**Authors:** Sen Chen

**Affiliations:** State Key Laboratory of Mechanics and Control for Mechanical Structures, Institute for Frontier Science, Nanjing University of Aeronautics and Astronautics, Nanjing 211100, China; chensen@nuaa.edu.cn

**Keywords:** liquid metals, biomimicry, interfaces, fluidity, smart materials, soft robotics, flexible electronics, neuromorphic computing

## Abstract

Liquid metals, particularly gallium-based alloys, uniquely combine fluidic compliance with metallic conductivity, which makes them ideal candidates for biomimetic design. Rather than treating biomimicry as the mere imitation of biological forms, we argue that liquid metal biomimicry should be understood as the realization of biological strategies through the intrinsic physics of fluidity and interfacial dynamics. This review organizes existing research within a hierarchical framework that couples physical liquidity, interface biology analogy, and functional emergence to explain how adaptive behaviors naturally arise from dynamic liquid metal systems. We examine representative systems across morphological and functional dimensions and contend that their true significance lies not in replicating nature but in addressing problems that conventional rigid materials cannot solve. Looking forward, we identify several transformative directions that collectively chart a roadmap toward truly intelligent and autonomous bioinspired systems. By bridging the physics of fluidity with the principles of biological adaptability, liquid metal biomimicry holds transformative potential for soft robotics, wearable electronics, neuromorphic computing, and biomedical engineering.

## 1. Introduction

Nature has evolved highly adaptive material systems through billions of years of evolution, enabling living organisms to continuously sense, respond, repair, and adapt to complex and dynamic environments. Unlike conventional engineering materials that generally rely on fixed structures and predefined functions, biological systems achieve remarkable robustness through dynamic interfaces, hierarchical organization, continuous energy dissipation, and multiscale feedback regulation [1,2]. These characteristics have inspired the rapid development of biomimetic materials [3], where biological structures, functions, and design principles are translated into artificial systems to achieve enhanced adaptability, intelligence, and multifunctionality. During the past decade, biomimicry has become an increasingly important strategy in materials science [4,5], driving innovations ranging from superhydrophobic surfaces inspired by lotus leaves to gecko-inspired adhesives [6], biomedical applications [7,8], and flexible electronics [9,10]. More recently, the focus of biomimetic research has gradually shifted from simply reproducing biological morphologies toward understanding and recreating the underlying physical principles that enable biological adaptability.

Among emerging adaptive materials, gallium-based liquid metals occupy a particularly unique position because they combine metallic conductivity with intrinsic fluidity under ambient conditions [11]. Unlike conventional metals, liquid metals possess exceptional deformability [12,13], high electrical and thermal conductivity [14,15], and a dynamically evolving oxide skin that continuously interacts with surrounding environments [16]. These properties have enabled extensive applications in flexible electronics [17,18], soft robotics [19,20], catalysis [21], and biomedical engineering [22]. During the past several years, the rapid development of liquid metal composites, ionically coupled systems, and reconfigurable conductive networks has further expanded the functional landscape of liquid metals beyond simple conductive fluids. Consequently, liquid metals are increasingly recognized not merely as structural or conductive materials, but as dynamic material platforms capable of continuously adapting their morphology, interfaces, and transport pathways in response to external stimuli. Beyond these engineering capabilities, liquid metals increasingly display behaviors that resonate with biological systems. A growing body of work has demonstrated life-like phenomena, including self-fueled liquid metal mollusks [23], amoeba-like locomotion [24], self-growing pseudopod-like locomotion [25], liquid metal cell [26], liquid metal ink sac [27], liquid metal artificial synapse [28], and bioinspired pupil reflex [29]. At the mechanistic level, electrically or chemically driven deformation enables autonomous shape reconfiguration and directional migration [19,30,31], while the repeated rupture and self-healing of the oxide skin resemble the adaptive remodeling of biological membranes that balance protection with selective permeability. In parallel, reversible oxidation–reduction reactions have enabled liquid metal systems to exhibit information storage behaviors reminiscent of biological polarization and depolarization processes [32]. Collectively, these observations indicate that liquid metals are no longer confined to conventional material response regimes, but are beginning to operate as adaptive systems governed by principles reminiscent of living matter [33]. From this perspective, liquid metal systems provide a compelling bridge between soft matter physics and biological adaptability, offering a unified framework for understanding how fluid interfaces, interfacial chemistry, and transport processes can collectively generate life-like functionality.

**Figure 1:**
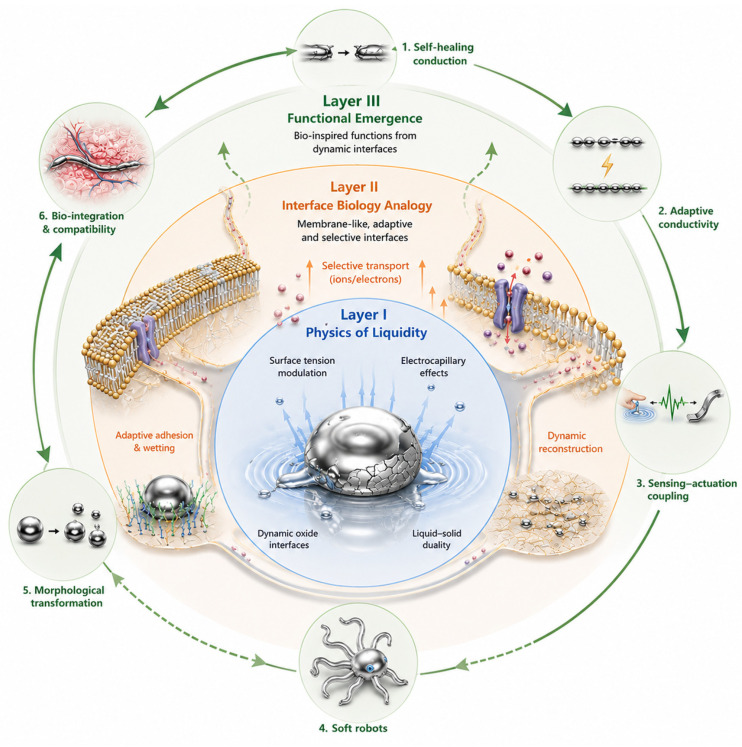
Liquid metal biomimicry: conceptual framework. The framework comprises three interconnected levels: Physics of Liquidity, Interface Biology Analogy, and Functional Emergence. Physical properties of liquid metals (Layer I) govern adaptive interfacial behaviors analogous to biological membranes (Layer II), giving rise to emergent functions (Layer III). Multidirectional interactions and feedback loops enable the system to adapt, self-regulate, and perform complex bio-inspired tasks.

In this review, we propose “liquid metal biomimicry” as a conceptual framework for understanding and designing adaptive liquid metal systems through biologically inspired principles, providing the first systematic synthesis that explicitly bridges the physics of fluidity with biological adaptability. Rather than defining biomimicry as the simple imitation of biological structures or motions, we argue that it should be understood as the realization of biological strategies through the intrinsic physics of liquid metals. As illustrated in Figure 1, this framework is organized into three interconnected levels. “Physics of Liquidity” establishes the fundamental physical basis through surface tension modulation, liquid–solid duality, electrocapillary effects, and dynamic oxide interfaces. Building upon these, “Interface Biology Analogy” interprets liquid metal interfaces as dynamic membrane-like systems capable of selective transport, adaptive adhesion, and continuous structural reconstruction. Their coupling ultimately gives rise to “Functional Emergence”, where self-healing conduction, adaptive conductivity, morphology transformation, sensing–actuation coupling, and soft robots arise as system-level behaviors through continuous feedback among physical forces, interfacial evolution, and environmental interactions. On this basis, we examine biomimetic systems across morphological and functional dimensions, from biomimetic locomotion to neuron-inspired memory, and argue that the central value lies not in superficial resemblance to life but in solving problems beyond the reach of conventional rigid systems. We also identify persistent challenges and outline future directions from programmable interfaces to cognitive liquid matter, charting a pathway toward liquid metal systems that deliver a practical impact in soft robotics, wearable electronics, and biomedical engineering.

## 2. From Biological Inspiration to Design Principles

Biomimicry has long served as one of the most productive paradigms for advancing materials science and engineering. Rather than directly copying biological structures, modern biomimetic research increasingly seeks to identify the fundamental design principles that enable living organisms to achieve robustness, adaptability, and multifunctionality. Throughout evolution, biological systems have developed highly efficient strategies for sensing environmental changes, regulating internal states, repairing structural damage, and optimizing energy utilization through dynamic interactions across multiple spatial and temporal scales. These capabilities emerge not from individual material properties but from the coordinated coupling between interfaces, structures, and functions operating far from thermodynamic equilibrium. Consequently, biomimicry has evolved from morphology-inspired design toward principle-inspired engineering, where the ultimate objective is to reproduce the adaptive mechanisms underlying biological intelligence instead of merely replicating biological appearances.

Liquid metals naturally provide an ideal platform for implementing this emerging biomimetic philosophy. Unlike conventional engineering materials that rely on fixed geometries and static functional architectures, liquid metal systems continuously evolve through the interplay between fluid mechanics, interfacial chemistry, electrochemical reactions, and external stimuli. Their ability to dynamically reconstruct conductive pathways, reversibly regulate interfacial properties, and autonomously adapt morphology places them remarkably close to the operational principles observed in living systems. Therefore, introducing biomimicry into liquid metal research should not be regarded as adding biological features onto an existing material platform. Instead, it represents a conceptual transition from designing static materials with predefined functions toward developing adaptive material systems capable of continuously interacting with and responding to their environments. Within this framework, nature serves not only as a source of inspiration for novel structures but also as a guide for understanding how intelligent behaviors can emerge from the coupling between physical laws, dynamic interfaces, and environmental feedback.

## 3. Representative Liquid Metal Biomimetic Systems and Applications

### 3.1. Morphology-Inspired Adaptive Systems

**Figure 2:**
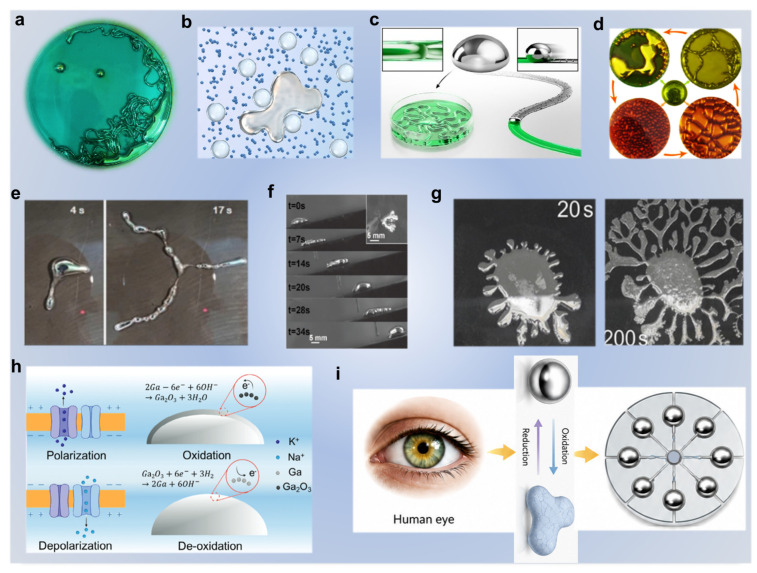
Representative liquid metal biomimetic systems and applications. (**a**) Self-growing serpentine movement of liquid metal induced by copper ions [25]. (**b**) Biomimetic chemotactic movement of liquid metal triggered by copper salt particles [35]. (**c**) Autonomous liquid metal droplets actuated by copper ion diffusion [36]. (**d**) Spontaneous dispersion and large-scale deformation of liquid metal induced by ferric ions [38]. (**e**) Amoeba-like movement of Al-eating liquid metal on graphite plates [39]. (**f**) Anti-gravity movement of liquid metal with worm-like morphology under an electric field [40]. (**g**) Fractal phenomena of liquid metal triggered by synergistic oxidation [41]. (**h**) Principle of liquid metal based flexible memory based on oxidation and de-oxidation inspired by cell membrane polarization and depolarization [32]. (**i**) Bioinspired adaptive pupil reflex based on liquid-metal shape-shifters for machine vision [29].

Owing to its superior fluidity and large surface tension, liquid metal systems readily exhibit rich morphological dynamics driven by interfacial oxidation, and external fields, enabling a variety of life-like motility behaviors. Early studies primarily focused on exploiting these intrinsic fluidic properties to generate biomimetic motions resembling those of simple organisms. As illustrated in Figure 2a, Chen et al. reported a self-growing serpentine movement of liquid metal driven by copper-ion-induced surface reactions [25], where anisotropic interfacial energy continuously generated directional growth resembling the crawling behavior of snakes. Unlike conventional robotic locomotion that relies on external mechanical actuation, the movement emerged spontaneously through self-sustained interfacial reactions, highlighting the ability of liquid metals to transform chemical energy directly into adaptive motion. This work provided one of the earliest examples showing that liquid metals can generate autonomous biomimetic behaviors solely through physicochemical reactions between gallium and related reactive ions. Following this concept, Wang et al. investigated the effect of anions on deformation of gallium-based liquid metal in copper salt solution [34]. Recently, as illustrated in Figure 2b,c, Ma et al. elucidated the leukocyte-like chemotactic locomotion of liquid metal [35] and Tang et al. proposed autonomous liquid metal droplets actuated by ion diffusion [36]. Fundamentally, these subsequent studies can be regarded as multidimensional investigations into the interfacial phenomena induced by the reaction between gallium and copper salt solutions, thereby significantly enriching the profound implications of this prototypical system. Future research is anticipated to provide an in-depth elucidation of the underlying mechanisms governing this phenomenon, as well as to explore its potential practical applications. In addition to copper ions, Fe ion-induced spontaneous dispersion further revealed that interfacial reactions could trigger large-scale morphological reconstruction rather than simple translation (Figure 2d). Despite being addressed in subsequent research [37], the profound implications and latent opportunities associated with this phenomenon remain largely unexplored.

The liquid metal–aluminum coupling system has attracted considerable attention due to its spontaneous self-propelled motion and continuous consumption of aluminum fuel [23], which resembles biological feeding and locomotion processes. In this system, the sustained consumption of aluminum induces asymmetric surface tension gradients and interfacial instability, enabling autonomous motion without external mechanical actuation. When further placed on a graphite substrate, the same system exhibits a more visually biomimetic behavior, namely amoeba-like crawling (Figure 2e) [39]. This transformation arises from the modified interfacial interaction between the liquid metal and the graphite surface, which alters the wetting state and mechanical confinement, thereby facilitating more compliant shape reconfiguration. Building upon this substrate-mediated interfacial tuning, the liquid metal evolves into a worm-like morphology capable of anti-gravity motion under an applied electric field (Figure 2f) [40], where electrohydrodynamic forces overcome gravitational constraints and sustain directional deformation. The underlying mechanism is strongly dependent on the coupled effects of electric field distribution, interfacial tension modulation, and substrate-mediated confinement, highlighting the importance of multi-field interactions in governing liquid metal dynamics. More intriguingly, when operated under similar interfacial conditions, the system can also generate fractal-like growth patterns widely observed in natural systems (Figure 2g) [41]. Such fractal evolution reflects a complex interplay between fluid dynamics, interfacial chemistry, and materials transport processes, leading to self-similar structural organization across multiple length scales. Although these behaviors remain fundamentally physics-driven rather than biologically regulated, they provide an important bridge between fluid mechanics and biological morphology, establishing a foundation for understanding how simple interfacial systems can give rise to life-like movement patterns. These phenomena not only deepen our understanding of nonlinear interfacial systems and the emergence of life-like behaviors from simple physicochemical interactions, but also hold promise for practical applications in future bioinspired functional materials. As fundamentally physics-driven processes rather than biologically regulated systems, they provide a conceptual bridge between fluid mechanics and biological morphology, offering a foundation for interpreting how complex motion patterns can arise from dynamic interfacial systems.

### 3.2. Function-Inspired Intelligent Devices

While morphological mimicry emphasizes resemblance in appearance, recent research has increasingly focused on reproducing biological functions through dynamic interfacial regulation. A representative example is the liquid metal flexible memory shown in Figure 2h, which draws inspiration from the reversible polarization and depolarization of biological cell membranes. Instead of relying on conventional semiconductor switching mechanisms, reversible oxidation and reduction of the liquid metal surface dynamically modulate electrical resistance, enabling stable and programmable memory states. This work represents an important conceptual transition from mimicking biological morphology to reproducing biological information-processing mechanisms through interfacial electrochemistry. A second representative example is the adaptive artificial pupil illustrated in Figure 2i, where electrically controlled oxidation and reduction regulate the deformation of liquid metal arrays to reproduce pupil reflexes observed in living organisms. Compared with conventional mechanically actuated optical systems, the liquid metal design offers continuous, reversible, and highly compliant shape modulation, providing a promising strategy for adaptive machine vision and intelligent optical devices.

Beyond these representative examples, similar design concepts have recently been extended to a broad range of emerging liquid metal-based systems, including self-healing electronics, adaptive conductors, neuromorphic devices, soft sensors, and reconfigurable circuits. In self-healing electronics, Li et al. demonstrated a universal strategy for self-healing materials via dynamic interfacial liquid metal coordination [12], while Han et al. developed conductive liquid metal–vitrimer composites for reconfigurable and recyclable flexible electronics [42], and liquid metal–elastomer composites have been designed for robust underwater electronics and stretchable RF electronics [43]. For adaptive conductors, Li et al. reviewed the structural engineering strategies for designing strain-insensitive conductors [44], which are enabled by the superior fluidity of liquid metals. In neuromorphic devices, Wan et al. proposed a biomimetic liquid metal synapse [28], and a liquid metal-based module has been developed to emulate the intelligent preying logic of flytrap [45]. Soft sensors leveraging hierarchical self-healing liquid metal architectures have achieved ultrasensitive strain sensing [46], where the embedded liquid metal microdroplets form a multiscale conductive network that spontaneously reconnects fractured pathways upon mechanical damage while electrochemical synergy accelerates healing through oxide regeneration. In addition, recyclable self-healing strain sensors based on liquid metal and Diels–Alder polymer have been reported for smart wearable applications [47]. Recently, Wang et al. achieved continuum tactile sensing via an amplified liquid metal interface [48]. Reconfigurable circuits have been realized through double-sided smart textile circuits and supercooled liquid metals for reconfigurable electronics [49]. In parallel, recent advances in additive manufacturing have opened up new avenues for constructing biomimetic liquid metal structures with unprecedented geometric complexity [50]. Collectively, these advances indicate that liquid metal biomimicry is gradually shifting from replicating biological forms toward implementing the adaptive functional principles that govern living systems, where continuously evolving interfaces replace rigid structural programming as the dominant design paradigm.

### 3.3. Mechanisms of Liquid Metal Biomimicry

**Figure 3:**
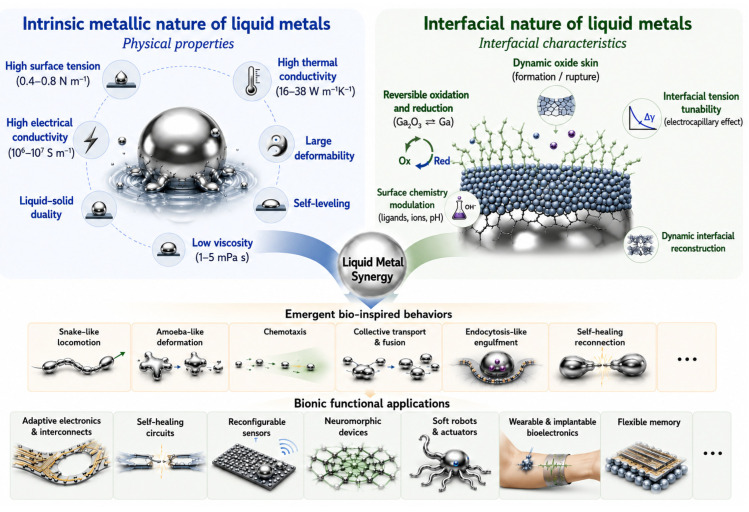
The synergy between intrinsic metallic nature and dynamic interfaces enables liquid metal systems to mimic life and empower intelligent technologies. Intrinsic metallic characteristics provide the physical basis for rapid shape transformation and charge transport. Dynamic interfacial characteristics continuously redefine environmental interactions. Their cooperation gives rise to adaptive behaviors and intelligent functionalities.

Although the biomimetic behaviors summarized in Figure 2 appear highly diverse, they originate from a common physicochemical foundation. As summarized in Figure 3, liquid metal biomimicry is governed by the synergistic interaction between intrinsic metallic properties and dynamically programmable interfaces. Intrinsic metallic characteristics, including high electrical conductivity, fluidity, metallic bonding, high surface tension, and excellent deformability, provide the physical basis for rapid shape transformation, efficient charge transport, and reversible structural evolution. These properties distinguish liquid metals from conventional polymers and hydrogels, allowing them to simultaneously function as structural, electrical, and responsive materials. Equally important are the dynamic interfacial characteristics of liquid metals. The spontaneous formation and reversible regulation of oxide layers and electric double layers, together with electrocapillary and electrochemical processes, continuously redefine interactions between liquid metals and their surrounding environments. Notably, electrocapillarity and electrochemically controlled oxidation represent two distinct yet complementary mechanisms for tuning interfacial tension. The former enables rapid and reversible modulation through electric-field-induced charge redistribution within the electric double layer, whereas the latter produces more persistent and larger-amplitude changes in interfacial properties through electrochemically driven oxide formation and reduction. Rather than acting as passive surface phenomena, these interfaces actively couple external electrical, chemical, and mechanical stimuli with internal structural evolution, ultimately giving rise to adaptive behaviors and intelligent functionalities. This unified physicochemical framework explains not only the morphology-inspired systems and function-inspired devices discussed above, but also provides a general design principle for future developments in soft robotics, adaptive electronics, flexible memories, bio-integrated devices, and intelligent human–machine interfaces. Understanding and engineering the cooperation between intrinsic metallic nature and dynamic interfacial regulation will therefore be central to the next generation of liquid metal biomimetic systems.

## 4. Beyond Mimicry: From Biological Appearance to Adaptive Intelligence

From the above discussion, it can be concluded that one of the earliest manifestations of liquid metal biomimicry is the reproduction of biological morphologies and movements. Owing to their intrinsic fluidity and dynamically reconfigurable interfaces, liquid metal droplets are capable of generating a remarkable variety of life-like behaviors under electrical, chemical, magnetic, or mechanical stimuli. Although these behaviors are entirely rooted in well-established physicochemical mechanisms rather than biological metabolism or genetic regulation, they nevertheless provide a powerful conceptual bridge between soft matter physics and biological function. They demonstrate how relatively simple interfacial laws, when coupled with nonlinear feedback and environmental coupling, can give rise to unexpectedly rich adaptive dynamics. In this sense, liquid metal systems shift from being viewed as passive conductive fluids toward being recognized as a class of active matter capable of emergent, morphology-driven behavior, reshaping how reconfigurable materials are conceptualized in both materials science and bioinspired engineering.

Nevertheless, the ultimate objective of liquid metal biomimicry should extend beyond reproducing either biological morphology or isolated biological functions. Biological intelligence emerges from continuous interactions among physical structures, chemical reactions, environmental feedback, and energy dissipation across multiple hierarchical levels. Accordingly, future liquid metal biomimicry should pursue the reconstruction of these adaptive principles rather than the imitation of individual biological phenomena. Whether a liquid metal system resembles a crawling organism or exhibits neuron-inspired electrical switching is, by itself, of limited scientific significance unless such behaviors provide measurable advantages in adaptability, robustness, efficiency, or multifunctionality. Therefore, the success of liquid metal biomimicry should not be evaluated by the degree of visual resemblance to living organisms, but by its ability to reproduce the underlying mechanisms that enable biological systems to continuously sense, regulate, and evolve under changing environments. In this sense, the field is gradually evolving from pursuing “similarity in form” toward achieving “similarity in principle,” where the ultimate benchmark is not whether liquid metals behave like living organisms, but whether biomimetic design enables fundamentally new capabilities that cannot be achieved with conventional engineering materials.

## 5. From Current Challenges to Future Adaptive Matter

**Figure 4:**
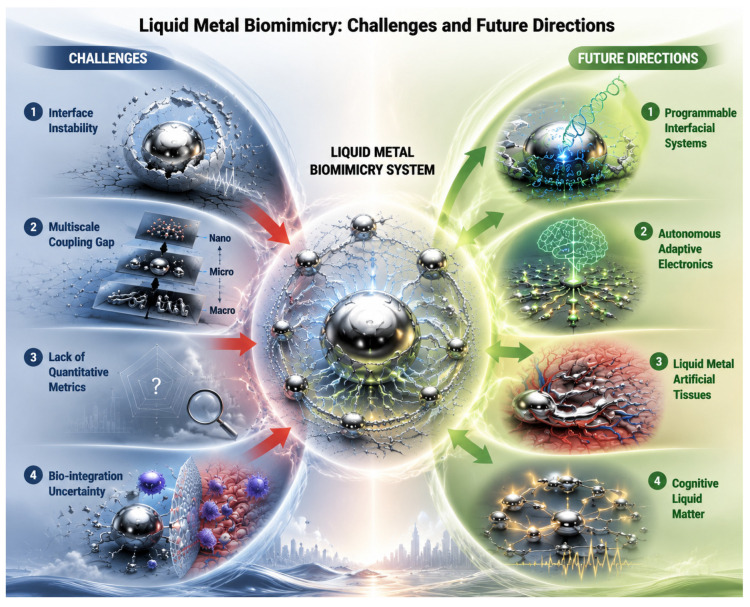
Liquid metal biomimicry: challenges and future directions. Key challenges include interface instability, multiscale coupling gaps, lack of quantitative metrics, and bio-integration uncertainty. Future directions encompass programmable interfacial systems, autonomous adaptive electronics, liquid metal artificial tissues, and cognitive liquid matter, charting a roadmap toward intelligent and autonomous bioinspired systems.

Despite remarkable progress over the past decade, liquid metal biomimicry remains at an early stage of development, and several fundamental scientific challenges continue to limit its broader advancement. As summarized in Figure 4, one of the most significant challenges lies in achieving precise control over dynamic liquid metal interfaces. Although oxide formation and reconstruction underpin many adaptive behaviors, these interfacial processes are often highly sensitive to local chemical environments, electrical conditions, and mechanical perturbations, making deterministic regulation difficult. The native oxide layer, typically stabilizing at approximately 3 nm, significantly alters interfacial behavior and complicates device fabrication and long-term operation. Furthermore, the adaptive behaviors observed in liquid metal systems span multiple length scales, ranging from atomic-scale interfacial reactions and microscale droplet interactions to macroscale structural evolution and device performance. Bridging these scales remains a formidable challenge, and current theoretical models are still insufficient to establish quantitative relationships between microscopic interfacial dynamics and macroscopic functional responses. These scales are not independent; interfacial chemistry at the nanoscale directly influences droplet-scale wetting and transport, which in turn determines device-level performance. Developing predictive multiscale frameworks requires integrating atomic-scale simulations (density functional theory, molecular dynamics), mesoscale models (phase-field, lattice Boltzmann), and continuum mechanics, with machine learning serving as a bridge across scales and in situ characterization providing validation data.

Equally important is the absence of standardized metrics for evaluating biomimetic performance. Existing studies frequently describe liquid metal systems using qualitative biological analogies, yet objective criteria for measuring adaptability, intelligence, or biological similarity remain largely unexplored. This lack of quantitative benchmarks not only hinders fair comparison across different studies but also obscures the pathway from laboratory demonstrations to practical applications. In parallel, we note that gallium-based liquid metals have demonstrated promising biocompatibility in numerous studies [51], with gallium ions naturally excreted through renal clearance and exhibiting antimicrobial properties. However, long-term bio-integration, chronic stability, and immune interactions continue to require systematic investigation before widespread biomedical translation becomes feasible. The gap between short-term in vitro biocompatibility and long-term in vivo performance remains substantial, and the degradation products of gallium-based alloys, their biodistribution, and their potential immunogenicity over extended periods are not yet fully understood.

Overcoming these challenges demands a shift from empirical material tuning to programmable adaptive liquid metal systems. Instead of only optimizing compositions or structures, future work should actively manipulate interfacial evolution via external stimuli to turn dynamic interfaces into tunable functional modules. This allows predictable, reversible control over mass transport, mechanical performance and electrical responses. Recent advances in continuous electrowetting and electrochemical redox synergistic strategies have demonstrated tunable and directional liquid metal manipulation, thereby expanding the design space for reconfigurable systems. Meanwhile, multiscale characterization, computational simulation and AI will help uncover adaptive behaviors originating from coupled physicochemical interactions.

Looking further ahead, liquid metal biomimicry is expected to evolve beyond individual devices toward intelligent adaptive matter capable of continuously interacting with complex environments. As illustrated in Figure 4, programmable interfacial systems may provide the foundation for autonomous adaptive electronics that can self-regulate their electrical and mechanical properties in response to environmental changes. The concept of “living liquid metal” composites, in which liquid metal is embedded with dormant bacterial spores, exemplifies this direction: such systems can self-heal, resist oxidation, and boost conductivity upon microbial awakening [52]. Simultaneously, the integration of liquid metals with hydrogels, living tissues, and bioactive materials may accelerate the development of bio-integrated electronic platforms and artificial tissue systems with enhanced physiological compatibility. More fundamentally, future research may explore whether liquid metal systems can acquire higher levels of adaptive functionality through history-dependent interfacial evolution, distributed conductive networks, and feedback-regulated material responses. Such systems would no longer behave as passive materials responding to isolated stimuli but as continuously evolving adaptive matter capable of sensing, processing, and responding to environmental information through coupled physical mechanisms. Achieving this vision will require close collaboration among materials science, physics, chemistry, biology, robotics, and artificial intelligence, ultimately transforming liquid metal biomimicry from an emerging research direction into a general design paradigm for next-generation bio-inspired smart materials.

## 6. Conclusions

In conclusion, liquid metal biomimicry represents a rapidly emerging field at the intersection of fluid physics, interfacial science, and biological inspiration, where the synergy between the intrinsic metallic nature of liquid metals and their dynamic interfaces enables a broad spectrum of biomimetic behaviors and functions, ranging from adaptive motion and morphological transformation to self-healing electronics, flexible memories, and bio-integrated systems. More importantly, the field is undergoing a conceptual transition from the imitation of biological forms toward the implementation of the adaptive principles that govern living systems. Despite these exciting advances, several fundamental challenges remain. Future progress will require rigorous quantitative metrics to evaluate biomimetic performance, as well as predictive multiscale frameworks capable of bridging nanoscale interfacial dynamics with macroscopic functionalities. Addressing these challenges will demand close collaboration across materials science, physics, chemistry, biology, and artificial intelligence. Looking ahead, the convergence of programmable interfaces, autonomous adaptability, and emerging cognitive capabilities may enable liquid metal systems to function as adaptive components in soft robotics, bioelectronics, and neuromorphic technologies. Ultimately, the significance of liquid metal biomimicry lies not in faithfully reproducing nature, but in translating biological strategies into practical technologies that overcome the limitations of conventional rigid materials and devices.

## Data Availability

No new data were created or analyzed in this study.
